# Dealing With Stress: A Review of Plant SUMO Proteases

**DOI:** 10.3389/fpls.2019.01122

**Published:** 2019-09-18

**Authors:** Rebecca Morrell, Ari Sadanandom

**Affiliations:** Department of Biosciences, Durham University, Durham, United Kingdom

**Keywords:** SUMO protease, SUMO cycle, Ubiquitin-like, Cysteine protease, Post-translational modification, Stress

## Abstract

The SUMO system is a rapid dynamic post-translational mechanism employed by eukaryotic cells to respond to stress. Plant cells experience hyperSUMOylation of substrates in response to stresses such as heat, ethanol, and drought. Many SUMOylated proteins are located in the nucleus, SUMOylation altering many nuclear processes. The SUMO proteases play two key functions in the SUMO cycle by generating free SUMO; they have an important role in regulating the SUMO cycle, and by cleaving SUMO off SUMOylated proteins, they provide specificity to which proteins become SUMOylated. This review summarizes the broad literature of plant SUMO proteases describing their catalytic activity, domains and structure, evolution, localization, and response to stress and highlighting potential new areas of research in the future.

## Introduction

SUMO (small ubiquitin-like modifier) proteins are critical for the normal function of eukaryotic cells; the protein is found in all eukaryotes from single-celled yeast *Saccharomyces cerevisiae* to all mammals and plants species. Deleting the only SUMO isoform, SMT3, from a yeast cell, causes a loss of cell viability (Meluh and Koshland, 1995; Giaever et al., 2002) and deletion in *Arabidopsis thaliana* of *sumo1sumo2-1* is embryonic lethal (Saracco et al., 2007; van den Burg et al., 2010), highlighting the critical role of SUMO in cell biology.

SUMO is an 11-kDa protein with one isoform in yeast and eight isoforms currently identified in *A. thaliana* through computational analysis (Novatchkova et al., 2004). In *Arabidopsis*, SUMO1, 2, 3, and 5 are expressed (Benlloch and Lois, 2018), but only SUMO1 and 2 are expressed at high levels (Castaño-Miquel et al., 2011). AtSUMO1 and 2 share 83% amino acid sequence identity and are orthologs of human SUMO2/3, based on sequence similarity. AtSUMO1/2 and human SUMO2/3 also have similar functions and are influenced by stress conditions. Conversely, AtSUMO3/5 are weakly expressed non-conserved isoforms. They are more distantly related to AtSUMO1/2 containing approximately 35% sequence similarity to AtSUMO1/2 (Castaño-Miquel et al., 2011).

SUMOylation is induced by heat, ethanol, drought stress, and oxidative stress; this is conserved in many species from *Arabidopsis, Drosophila*, and *Caenorhabditis elegans* to humans (Saitoh and Hinchey, 2000; Kurepa et al., 2003; Augustine et al., 2016). Indeed, there are many evolutionarily conserved SUMO targets and processes that SUMO is involved in throughout different species. Global proteomic studies in *C. elegans* have identified over 800 SUMO targets; based on these targets, *Arabidopsis* is predicted to have 5660 SUMOylated proteins. The breadth of SUMOylation is on par with other major PTMs (post-translational modifications) like phosphorylation and ubiquitylation (Drabikowski et al., 2018; Millar et al., 2019).

In addition to responding to stress, SUMO is also implicated in many essential cellular processes (Hannoun et al., 2010). Drabikowski et al. (2018) undertook a comprehensive analysis of all the proteins SUMOylated in *C. elegans*. They identified SUMOylated protein targets including proteins involved in genome stability, cell cycle progression, chromatin maintenance and modification, transcription, translation RNA splicing, and ribosome biogenesis. Many identified proteins were non-nuclear localizing in the mitochondria or extracellular matrix. Cytosolic proteins include proteasomal, ribosomal, metabolism, signaling, cell morphology, and motility (cytoskeleton, microtubules, intermediate filaments, and proteins connecting the cytoskeleton to the plasma membrane) proteins. In addition, transport and vesicular transport proteins were identified. The group predicted that at least 15–20% of the eukaryotic proteome can be SUMOylated and suggested that SUMO functions in three main areas: regulation of activity of individual proteins, biogenesis of macromolecular complexes, and SUMO-directed proteasomal degradation (Drabikowski et al., 2018).

A similar study was carried out by Rytz et al. (2018) looking into SUMOylated proteins in *Arabidopsis* found over 1000 SUMOylated targets; many of which were nuclear targeted. The proteins identified included major transcription factors, coactivators/repressors, and chromatin modifiers connected to abiotic and biotic stress defense (Rytz et al., 2018).

The SUMO cycle is likened to ubiquitin due to the similarities in the biochemical steps that catalyze SUMO conjugation and deconjugation of protein substrates (Kerscher et al., 2006). SUMO has a similar 3D structure to ubiquitin, adopting the signature β-grasp fold (characterized by β-sheet with five anti-parallel β-strands and a single helical element between strands β-4 and β-5) but only shares 20% sequence similarity with ubiquitin. SUMO is longer than ubiquitin and has a longer disordered C-terminal tail, which requires processing (Bayer et al., 1998). Like ubiquitin, SUMO can form polySUMO chains of multiple SUMO moieties attached to one lysine or a single-SUMO molecule can be attached to a single lysine residue.

**Figure 1:**
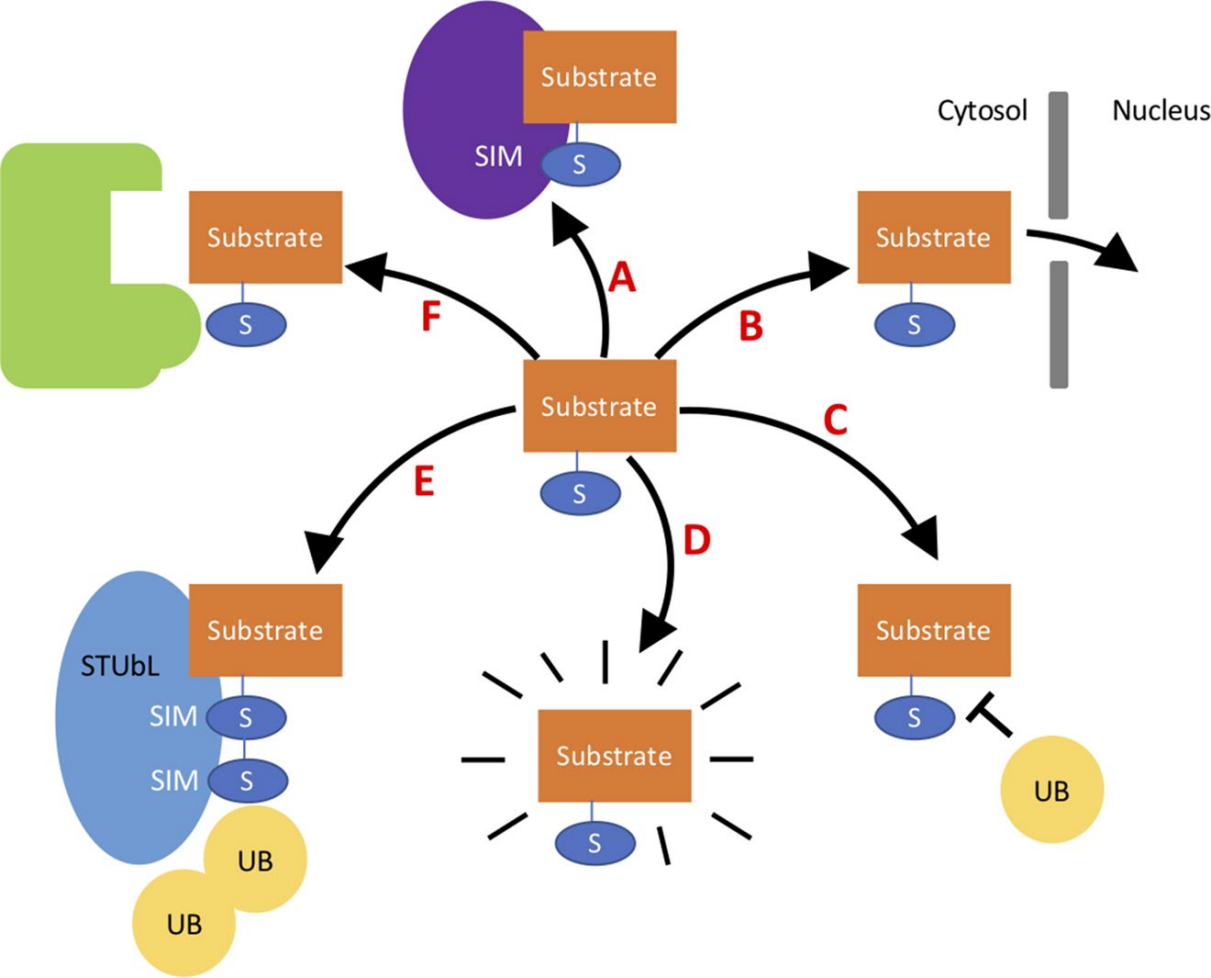
A variety of the different effects SUMO can have on a target substrate. A- SUMO can aid interaction with proteins containing a SIM site. B- SUMO can change the cellular localization of a protein—for example, directing the protein to the nucleus. C- SUMO can protect substrates from degradation by blocking lysine residues in substrates that may be ubiquitinated. D- SUMO binding to a protein can alter its structure activating the protein. E- SUMOylated proteins can signal to STuBL proteins to target for degradation *via* ubiquitination. F- SUMO can block interaction with proteins by blocking binding sites.

Ubiquitin conjugation to substrate proteins targets them for degradation; conjugation of SUMO can aid the substrate protein for degradation through STUbLs (SUMO-targeted ubiquitin ligases). STUbLs are a novel class of ubiquitin E4 ligases that target proteins with polySUMO chains, ubiquitinating the proteins resulting in degradation. STUbLs have been identified in plants but await biochemical analyses (Elrouby et al., 2013). However, unlike ubiquitin, SUMOylation can have numerous other effects on target proteins, protecting a protein from degradation by protecting lysine residues prone to ubiquitylation, changing its localization, or altering the protein–protein interaction or protein–DNA interactions (Johnson, 2004); the effects of SUMOylation are summarized in **Figure 1**. The protein–protein interaction can occur *via* a non-covalent bond that forms with proteins harboring SIM sites (SUMO-interacting motifs). SIMs are characterized by hydrophobic residues flanked by acidic residues or residues that can be phosphorylated. Alternatively, SUMO can prevent interactions by masking partner-binding sites.

The outcome of SUMOylation is largely target dependent and altered by the location and number of SUMO substrates on a target. Indeed, target protein substrates can have single SUMO monomers covalently attached to a lysine, multiple SUMO monomers attached to multiple lysines, or a polySUMO chain from one lysine. This results in a high complexity of different SUMO patterns that can form on one protein altering the molecular consequences of the SUMOylation. For example, the SUMOylation at one site may stabilize the protein by protecting the lysine from ubiquitination, and the SUMOylation at a second site may promote interaction with a protein harboring a SIM site that usually the substrate does not interact with. Despite the complexity of SUMO modification target proteins can experience, it has been observed that, commonly, only a small percentage of target proteins are SUMO modified at a given time entitled the “SUMO enigma” by Hay (2005).

## SUMO Cycle

**Figure 2:**
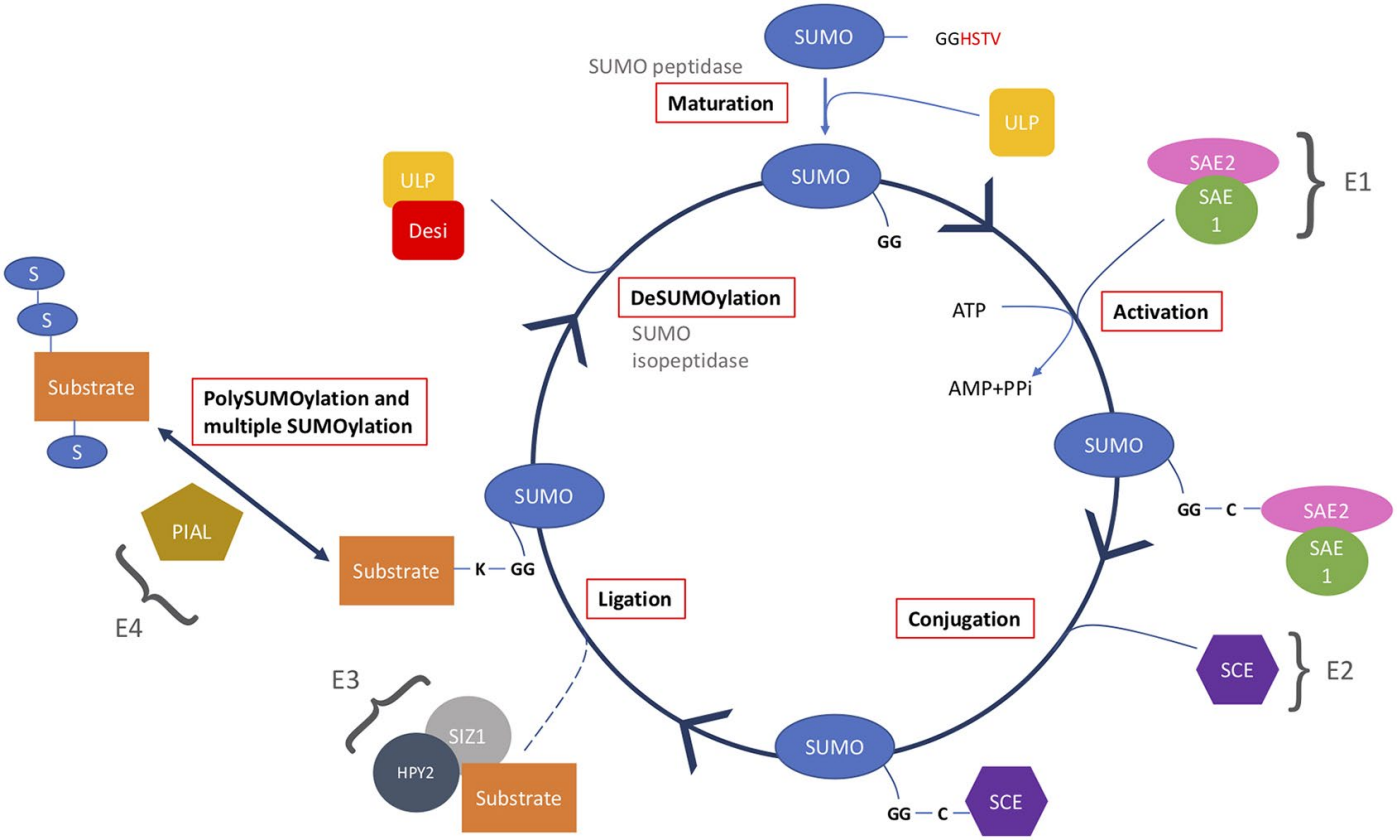
The SUMO cycle starts with maturation of immature SUMO by cleaving off the C-terminus using a SUMO peptidase. Mature SUMO is then activated using ATP and a heterodimer of SAE1 and SAE2. The SUMO is then passed to SCE in a conjugation step, and using a SUMO ligase is ligated onto the substrate. This substrate can then be SUMOylated at more than one SUMO site or form a polySUMO chain. Lastly, in the deSUMOylation step, SUMO is removed from the substrate using a SUMO isopeptidase to generate free SUMO.

SUMOylation is a highly dynamic cyclical process with SUMO changing between conjugated and non-conjugated free SUMO forms. SUMO conjugates are dynamic, changing during the cell cycle and in response to stimuli. The SUMO system accomplishes accurate, rapid, specific responses to stimuli. It has a series of biochemical steps, which are similar to ubiquitination, and involves activation, conjugation, and ligation (**Figure 2**).

Firstly, mature SUMO is generated by a SUMO peptidase cleaving 10 amino acids, exposing a carboxyl-terminal diglycine motif (Johnson, 2004). SUMO is activated by an E1 activation heterodimer of SAE1/2 (SUMO-activating enzyme). SAE1 has two isoforms SAE1a and SAE1b; either can be used to create the E1 heterodimer with SAE2, using ATP the complex forms SUMO-AMP. The AMP is released from the SUMO-AMP resulting in the formation of a high-energy thioester bond, between the sulfhydryl group of the catalytic cysteine residue in SAE2 and the carboxyl group of glycine in SUMO.

Activated SUMO is transferred from the SAE2 to a cysteine residue in SCE1 (SUMO conjugating enzyme), an E2 conjugation enzyme, in a transesterification reaction to form SUMO-SCE1 thioester complex. This complex catalyzes the reaction of SUMOylation onto a lysine in the target substrate *via* an isopeptide bond between the SUMO carboxyl terminal glycine and the ε-amino group of lysine (K). The lysine, typically, is part of the SUMOylation consensus motif *ψ*KXE/D; *ψ* denotes a large hydrophobic residue, K the acceptor lysine, X any amino acid, and E/D glutamate or aspartate.

SUMO E3 ligases are SCE1-interacting proteins that also help aid the transfer of SUMO from SCE1 to the target substrate. There are two identified E3 ligases in *Arabidopsis* SIZ1 (SAP and MIZ1) and HPY2 (high ploidy2), these complex proteins require a number of domains including nuclear localization and SUMO-interacting domains. However, SUMOylation of a target residue can occur without the presence of E3 ligases; it is as yet unclear how essential E3 ligases are.

An additional catalytic step has been identified involving E4 ligases. These ligases form SUMO chains; two E4 ligases have been identified in *Arabidopsis* PIAL1 and 2 (protein inhibitor of activated STAT-like 1/2).

Finally, SUMOylation is a cyclical process due to SUMO proteases, which carry out two main functions in the SUMO system. First, they cleave SUMO from target substrates, providing a pool of free SUMO, making SUMO a reversible modification (isopeptidase activity). Secondly, they mature newly synthesized SUMO by cleaving a c-terminal peptide from the immature SUMO (hydrolase/peptidase activity). These sources of SUMO are believed to be critical in the SUMO cycle as the cellular pools of unconjugated SUMO are very low (Johnson, 2004). In yeast, there are two SUMO proteases ULP1 and 2 (ubiquitin-like protease 1/2); the *ulp1-1* mutant is lethal, demonstrating the critical role of SUMO proteases in cell function.

In the ubiquitin system, it is assumed that the ubiquitin E3 ligases provide the specificity in the system due to the large number of E3 ligases. However, in the SUMO system, few SUMO E3 ligases have been identified. Conversely, a relatively larger number of SUMO proteases have been identified, which display specificity to the target proteins suggesting that they may provide specificity in the SUMO system (Chosed et al., 2006; Colby et al., 2006; Yates et al., 2016; Benlloch and Lois, 2018).

## Cysteine Proteases

There are four major classes of proteases in plants, described in the MEROPS database: cysteine-, serine-, aspartate-, and metallo-proteases, with the protease superfamily comprising 2% of coding genes in plants (Hou et al., 2018). All identified SUMO proteases are cysteine proteases. Cysteine proteases are named after the cysteine residue in their active site which is used as a nucleophile for the formation of an acyl intermediate during proteolytic cleavage (Rawlings et al., 2008). Cysteine proteases are often specialized proteases and are widespread in eukaryotes and in plants, with most species having many types of cysteine proteases. The cysteine proteases are important in plants due to their functions in many processes from seed germination to plant senescence. Environmental cues trigger changes in the proteases enabling the plant to react to stimuli including in response to biotic and abiotic stresses. In plants, cysteine proteases are typically found in lytic vacuoles (Roberts et al., 2012). The superfamily of cysteine proteases is highly conserved and has diversified greatly (Gillies and Hochstrasser, 2012).

### SUMO Proteases Belong to Different Classes of Cysteine Proteases

Previously, the SUMO proteases have belonged to the same protein superfamily, the ULP superfamily, identified due to their similarity to yeast ULP1/2 proteases. Currently, eight ULPs have been predicted in *Arabidopsis*; six have been characterized as SUMO proteases. The active site has a characteristic papain-like fold found in all ubiquitin-specific and UBL (ubiquitin-like)-specific cysteine proteases (Mukhopadhyay and Dasso, 2007; Hickey et al., 2012).

However, two new classes of SUMO proteases have since been identified. Shin et al. (2012) identified a novel type of SUMO protease in mouse; the two proteins were named DeSI1 (deSUMOylating isopeptidase1) and DeSI2, which lacked sequence similarity to ULP enzymes. Orosa et al. (2018) identified eight putative DeSI proteases in *Arabidopsis* based on sequence similarity to human DeSI1/2 and functionally characterized one protein, DeSI3a. Another SUMO protease recently identified in humans is called USPL1 (ubiquitin-specific protease-like1) (Schulz et al., 2012); currently, however, no homologues have been identified in *Arabidopsis*.

The three families of SUMO proteases identified are cysteine proteases. The cysteine proteolytic enzymes all contain a cysteine residue that acts as a nucleophile at the heart of the catalytic site triad or dyad capable of breaking the thioester bond between the SUMO and target protein. Also present in the active site in addition to the catalytic cysteine is a histidine that functions as a general base, and additionally, in some cases, there is an extra base that is required for stability. The orientation of the cystine, histidine, and stabilizing amino acid can differ. The SUMO proteases identified in yeast and *Arabidopsis* are listed in **Table 1**.

**Table 1:** List of currently identified SUMO proteases in yeast and *Arabidopsis*, giving all their known names, TAIR accession number, cysteine protease classification, and tissue expression.

Species	Name	Cysteine protease clan	Cysteine protease family	TAIR accession	Tissue expression
***Saccharomyces cerevisiae***	ULP1	CE	C48	NA	NA
	ULP2	CE	C48	NA	NA
***Arabidopsis thaliana***	OTS1 (ULP1d)	CE	C48	At1g60220	Root tissue, shoot vasculature of seedlings, developing flowers, wounding sites
	OTS2 (ULP1c)	CE	C48	At1g10570	Root tissue, shoot vasculature of seedlings, petioles, filaments, wounding sites
	ESD4	CE	C48	At4g15880	Seedlings, leaves, shoots, flowers and roots
	ELS1 (ULP1a)	CE	C48	At3g06910	Ubiquitously high levels in root vasculature tissue and flowers
	ELS2 (ULP1b)	CE	C48	At4g00690	Uncharacterized
	FUG1	CE	C48	AT3G48480	Uncharacterized
	SPF1/ASP1/ULP2like2	CE	C48	At1g09730	Ubiquitous in seedlings newly developing leaves and the tips of the roots. Also present in embryo sacs, inflorescences, anthers, and developing seed with intermediate expression levels in stems and rosette leaves
	SPF2/ULP2like1	CE	C48	At4g33620	Leaves, vasculature, inflorescences and maternal floral tissues, stems, cauline leaves, rosette leaves, and middle-length siliques
	Desi 1	CP	C97	At3g07090	Uncharacterized
	Desi 2A	CP	C97	At4g25660	Uncharacterized
	Desi 2B	CP	C97	At4g25680	Uncharacterized
	Desi 3A	CP	C97	At1g47740	Not known
	Desi 3B	CP	C97	At2g25190	Uncharacterized
	Desi 3C	CP	C97	At5g25170	Uncharacterized
	Desi 4A	CP	C97	At4g17486	Uncharacterized
	Desi 4B	CP	C97	At5g47310	Uncharacterized

Surprisingly, the three families of SUMO proteases currently identified (ULP, DeSI, and USLP1) are all members of different clans, which show evolutionary relationships between a broad number of proteases, despite all being SUMO proteases. The different clan classifications are due to the different amino acids and the order of the amino acids required for the active site. Within the clan, the proteases are further characterized into families which shows a statistically significant relationship in amino acid sequence to at least one other family member. The ULP proteases are members of the CE cysteine clan, further characterized into the C48 cysteine protease family. The CE clan is characterized as having catalytic triad with residues in the order histidine, glutamine (or asparagine), and then cysteine. The DeSI proteases are from the CP clan in the C97 cysteine protease family. The CP clan has a catalytic dyad composed of histidine and cysteine: in the DeSI active site, no third residue is required to orientate the histidine ring (Suh et al., 2012).The third type of SUMO protease USPL1 is a clan CA C98 cysteine protease family member (Rawlings et al., 2018). The CA clan is characterized as having the catalytic triad in the opposite orientation to that of CE clan with the residues cysteine, histidine, and asparagine (or Aspartic acid). However, all the proteases contain a papain fold, which characterizes all ubiquitin-specific and UBL-specific cysteine proteases characterized so far (Gillies and Hochstrasser, 2012).

### Proteolytic Mechanism of Cysteine Proteases

Cysteine proteases are characterized by containing a nucleophilic cysteine thiol that is responsible for the peptide bond attack providing the mechanism of proteolytic activity. The adjacent histidine, which acts as a general base, donates a proton to the cysteine residue to enhance the nucleophilicity. The cysteine’s anionic sulfur attacks the carbonyl carbon of the substrate, producing the first tetrahedral thioester intermediate in the reaction, releasing an amine or amino terminus fragment from the substrate. Additionally, the histidine residue in the catalytic triad is restored to its deprotonated form, and an intermediate is formed. The thioester bond is hydrolyzed, to produce a carboxylic acid moiety from the remaining substrate fragment helping form the oxyanion hole.

The catalytic action of the protease depends on the clan. In the CA clan, including USPL1, catalysis is caused by an acyl-enzyme intermediate, a glutamine amino acid that helps form the oxyanion hole that contains an electrophilic center. The electrophilic center helps stabilize the tetrahedral intermediate, and asparagine orientates the imidazolium ring of the catalytic histidine.

Whereas the ULP proteases are clan CE, the catalysis uses glutamine that, as in clan CA, helps form the oxyanion hole and glutamine that has a similar role to asparagine in CA clan of orientating the imidazolium ring of histidine. Additionally, an asparagine helps stabilize the histidine in the catalytic dyad. Many of the CE proteases show a preference for cleaving diglycine which may be due to a tryptophan following the catalytic histidine (Golubtsov et al., 2006). The tertiary structure for some of the CE proteases has been solved showing the active site is located between two structural subdomains, one being the beta barrel and the second a helical bundle.

In contrast, the DeSI proteases are the only yet identified members of the CP clan, which was identified when the crystal structure was solved (Suh et al., 2012). Unlike clan CA and CE proteases, there is no third active residue to orient the histidine ring; asparagine has been identified as a structurally important amino acid, but it has been shown to not interact with the catalytic histidine. The DeSI1 proteins forms a homodimer; this provides a prominent surface groove between the two monomers, similar to clan CA with the active site being located between a helix and a beta barrel, with the active sites directed toward the groove (Suh et al., 2012).

## Identification of the SUMO Proteases in Plants

ULP1 (ubiquitin like protease 1) was the first isolated SUMO protease in an activity-based screen of yeast *S. cerevisiae* (Li and Hochstrasser, 1999). ULP1 has an essential role in the G2/M phase of the yeast cell cycle. It was identified by expressing yeast enzymes from a genomic DNA library in *Escherichia coli*. The transformed *E. coli* was also transformed to express a substrate composed of histidine-tagged ubiquitin fused to HA-tagged SMT3. Running the bacterial extracts on SDS-PAGE gels could determine if cleavage after the diglycine motif had occurred between the SMT3 and ubiquitin.

ESD4 (early in short days4) was the first characterized SUMO protease in *Arabidopsis*, firstly identified by Reeves et al. (2002) by conducting a general mutagenesis study. ESD4 was found due to its early flowering phenotype and the reduced mRNA abundance of floral repressor FLC (floral locus C). However, it was not identified as a SUMO protease until Murtas et al. (2003) sequenced ESD4 and searched databases for proteins with similar sequence homology. The search identified similar proteins including human SENP1 (sentrin-specific protease 1), yeast ULP1, and mouse SMT3IP1. The protein similarity was approximately 200 amino acids in the C-terminus forming the active site of a cysteine protease. To confirm ESD4 as a SUMO protease, ESD4 was purified and its peptidase activity monitored *in vitro*. Furthermore, the activity of ESD4 was blocked by thiol reagent (cysteine protease inhibitor) *N*-ethylmaleimide (NEM), which is also inhibited yeast ULP1. However, ESD4 was not inhibited by ubiquitin aldehyde which inhibits deubiquitinating protease. Additionally, a mutant of ESD4, with the catalytic cysteine in the catalytic triad mutated to a serine, was assayed for deSUMOylation activity and was found to be inactive. Finally, the double glycine motif at the C-terminus of the SUMO that linked to FLC was mutated to two alanine residues. No cleavage was detected by ESD4 between the SUMO and FLC. *Arabidopsis esd4-1* mutants also have reduced levels of free SUMO and an increased abundance of SUMO conjugates.

Kurepa et al. (2003) identified *A. thaliana* SUMO proteases by their sequence similarity to yeast ULP1. The study used BLAST to identify *Arabidopsis* proteins with sequence similarity to animal and yeast ULP1 catalytic domains. The catalytic domain is a conserved region of 200 amino acids, which surrounds a triad of histidine, aspartate, and cysteine residues. The search found 12 genes which were further classified into three subfamilies, with two subfamilies more related to yeast ULP1 and the third more similar to yeast ULP2. Of the SUMO proteases identified by Kurepa et al. (2003), they noted on the lack of similarity in the proteins outside of the ULP1 catalytic domain; hypothesizing this may provide substrate specificity.

ELS1 (ESD4-like SUMO protease1) (which was identified by Kurepa et al. (2003) and named ULP1a) was characterized due to its sequence similarity to ESD4. ELS1 contains the same ULP1-catalytic domain as in ESD4 and ULP1. The SUMO activity was also assayed *in vitro* in the same assay as ESD4, using purified ESL1 to cleave SUMO from an extension. As with ESD4, the SUMO protease activity was blocked with NEM and when the catalytic cysteine was mutated to serine. Despite the sequence homology between ESD4 and ELS1, they do not show functional redundancy (Hermkes et al., 2011).

ELS2 was identified in the initial blast search performed by Kurepa et al. (2003) that also identified ELS1, named ULP1b. FUG1 (fourth ULP gene) was identified by Lois (2010) using human SENP1 and was shown to have an expressed sequence tag. It was classified to fourth ULP gene class due to its different phylogeny. Both proteases have been identified *via* bioinformatic techniques but are yet to be functionally addressed.

OTS1 and 2 (overly tolerant to salt 1/2) were initially identified in *Arabidopsis* in the screen by Kurepa et al. (2003) and named ULP1d and ULP1c, respectively. Chosed et al. (2006) and Colby et al. (2006) demonstrated that OTS1 and OTS2 had *in vitro* SUMO protease activity. Chosed et al. (2006) designed an assay with SUMO with an HA (hemagglutinin) tag fused to the diglycine residues and incubated this substrate with purified SUMO proteases. If the SUMO-HA is cleaved, the product runs faster on an SDS/PAGE gel and can be identified. The assay demonstrated that OTS1 and OTS2 can cleave SUMO1 and SUMO2 to produce mature SUMO. They then tested for isopeptidase activity by purifying RanGAP (RanGAP [GTPase-activating protein]) substrate modified by various recombinant GST-SUMO proteins. Both OTS1 and OTS2 were capable of cleaving various SUMO variants from RanGAP. In a similar approach, Colby et al. (2006) also expressed purified OTS1 and OTS2 and assayed for isopeptidase activity using *in vitro* SUMOylation. Initially, they tested AtSUMO1, 2, and 3 conjugated to ScPCNA (proliferating cell nuclear antigen). OTS1 and OTS2 cleaved SUMO1 and SUMO2 from ScPCNA. Colby et al. (2006) also used a similar assay to determine that OTS1 and OTS2 in addition to possessing isopeptidase activity also possess peptidase activity capable of maturing SUMO. Conti et al. (2008) demonstrated that *ots1ots2-1* mutants had increased levels of SUMO conjugates.

SPF1 and SPF2 (SUMO protease related to fertility 1/2) were initially identified by Novatchkova et al. (2004) and called ULP2-like-2 and ULP2-like-1, respectively. They were identified by searching the *Arabidopsis* genome for a protein that was most similar to the yeast query protein, ULP2. The proteins identified however were not characterized as a SUMO proteases until 2017. Two groups characterized the proteins: Liu et al. (2017a) renamed the proteins SPF1 and SPF2, Kong et al. (2017) renamed the ULP2-like-2 ASP1 (*Arabidopsis* SUMO protease1). Kong et al. (2017) confirmed *in vitro* that SPF1 has endopeptidase activity by incubating purified SPF1 with SUMOylated FLC and demonstrating that SUMO was cleaved by WT (wild-type) SPF1 but not by a mutation to the cysteine catalytic site or when NEM was added to the incubation. Additionally, they showed that both SPF1 and SPF2 can process immature SUMO to the mature form, and both single and double mutant knockouts have higher levels of SUMO conjugates. Kong et al. (2017) showed that the number of SUMO conjugates remain higher after heat shock in the *spf1-1* mutant.

Unlike the ULP proteins, DeSI1 was not identified from yeast as it does not contain a homologue. Originally, the DeSI proteins were identified as PPPDE (peptidase-permuted papain fold peptidases) of dsRNA (double-stranded RNA) viruses and eukaryotes. The DeSI proteins were identified in mice by Shin et al. (2012) using BZEL (BTB-ZF protein expressed in effector lymphocytes) in a yeast two-hybrid screen as bait. It was predicted to function as a deubiquitinating peptidase, but no activity had been reported. DeSI1 was unable to deubiquitinate ubiquitinated BZEL but was capable of deSUMOylating SUMOylated BZEL; additionally, DeSI1 was capable of cleaving polymeric SUMO2/3 from targets in mouse. Mutating the catalytic cysteine in DeSI1 abolished the deSUMOylation capabilities of DeSI-1. However, the DeSIs do not have SUMO-processing peptidase activity (Shin et al., 2012, Suh et al., 2012).

Orosa et al. (2018) searched for *Arabidopsis* homologues using the mammalian DeSI1 active site as a search criteria identifying eight putative *Arabidopsis* DeSI proteins. One protein, named DeSI3a, was purified and assayed *in vitro* for deSUMOylation activity, compared to the same protein with the catalytic cysteine mutation to serine, which was incapable of deSUMOylation. DeSI3a WT (wild type) showed cleavage of the isopeptide-linked SUMO; the mutated DeSI3a did not show activity. Additionally, DeSI3a was shown to specifically reduce higher molecular weight SUMO-conjugated isoforms of the kinase domain of FLS2 (flagellin-sensitive2), which the mutated form of DeSI3a was unable to (Orosa et al., 2018).

Schulz et al. (2012) were encouraged to search for novel SUMO proteases due to the low number of identified SUMO proteases and the small number of different families of SUMO proteases compared to ubiquitin proteases. They used an activity-based search with suicide substrates that irreversibly cross-link with SUMO proteases. This technique has been used for the identification of ubiquitin proteases; they purified HA-tagged SUMO ligated to vinylmethylester and incubated the SUMO with human cell lysates. The HA-tagged proteins were immunopurified from the lysate and analyzed with mass spectrometry. This led to the identification of USPL1, which, when the catalytic cysteine was mutated to the serine, USPL1 was no longer capable of binding to the SUMO. USPL1 was shown to interact with SUMO2, but not ubiquitin, to have some peptidase activity, and it shows some chain editing activity. Fluorescence-tagged USPL1 was found exclusively in Cajal bodies which are in the nucleus, associated with mRNA processing, and are highly dynamic, changing in number, size, and composition during cell cycle, development, and stress (Cioce and Lamond, 2005; Nizami et al., 2010). Interestingly, RNAi-mediated knockdown of USPL1 affected proliferation and COILIN localization; however, the phenotype was rescued by both USPL1 and the mutated form of USPL1, suggesting it has other functions (Schulz et al., 2012; Hutten et al., 2014). USPL1 highlights the importance of characterization of proteases, as USPL1 was originally misannotated as an ubiquitin protease due to sequence similarity.

**Figure 3:**
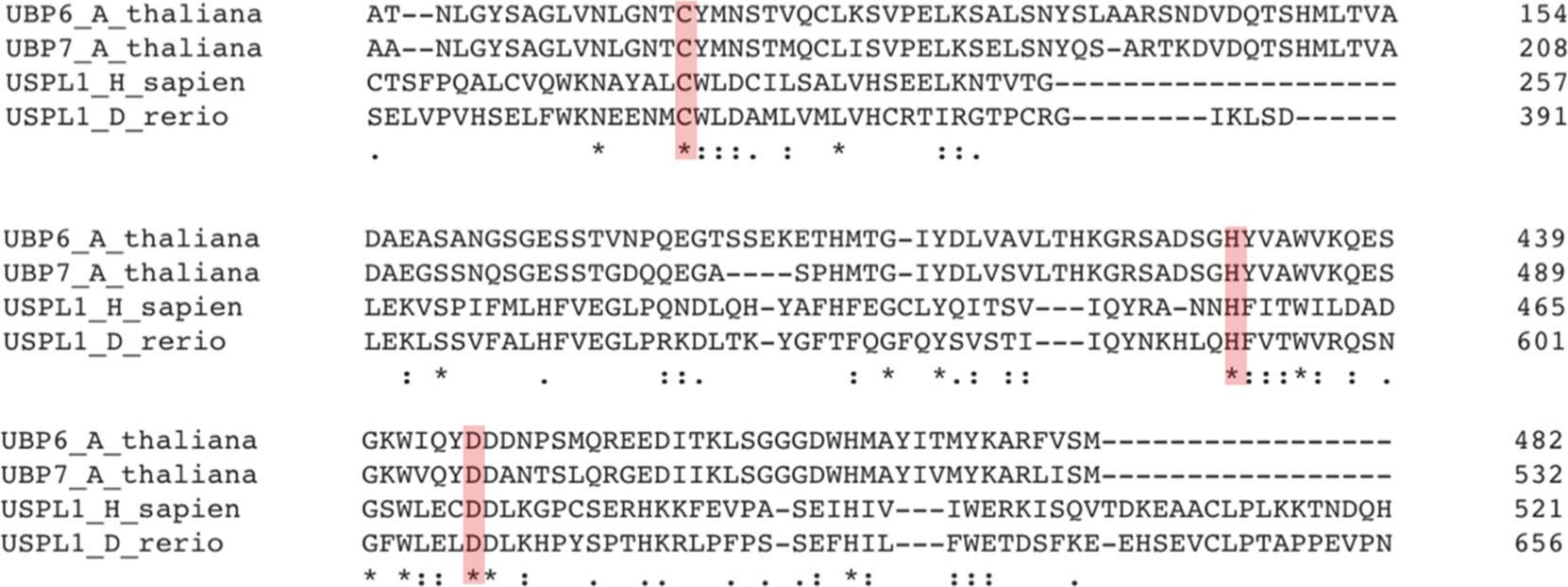
*Arabidopsis* UBP6 and UBP7 may be a distant USPL1 homologue. Alignment of human USPL1, zebrafish USPL1, *Arabidopsis* UBP6, and *Arabidopsis* UBP7. The red boxes highlighting the conserved residues indicate the catalytic triad.

Following previously outlined techniques to identify SUMO proteases in *Arabidopsis*, the catalytic site of USPL1 was blasted into the *Arabidopsis* genome. Two proteases were identified as potential matches: UBP6 and UBP7 (ubiquitin-specific protease6/7) shown in **Figure 3**. Both these proteases have already been identified as ubiquitin proteases through bioinformatic techniques. Moon et al. (2005) identified the protease as an interacting partner with CAM2 (Calmodulin2). However, they were unable to demonstrate in *E. coli* that UBP6 was capable of cleaving ubiquitin from substrates. It can only be speculated that UBP6 and its close homologue UBP7 are SUMO proteases currently. It will require functional characterization to prove their SUMO proteolytic activity. If, however, it is shown that UBP6/7 are SUMO proteases, it will be the third identified cysteine protease family present in *Arabidopsis*; there may be more yet.

## Domains and Structures of SUMO Proteases

**Figure 4:**
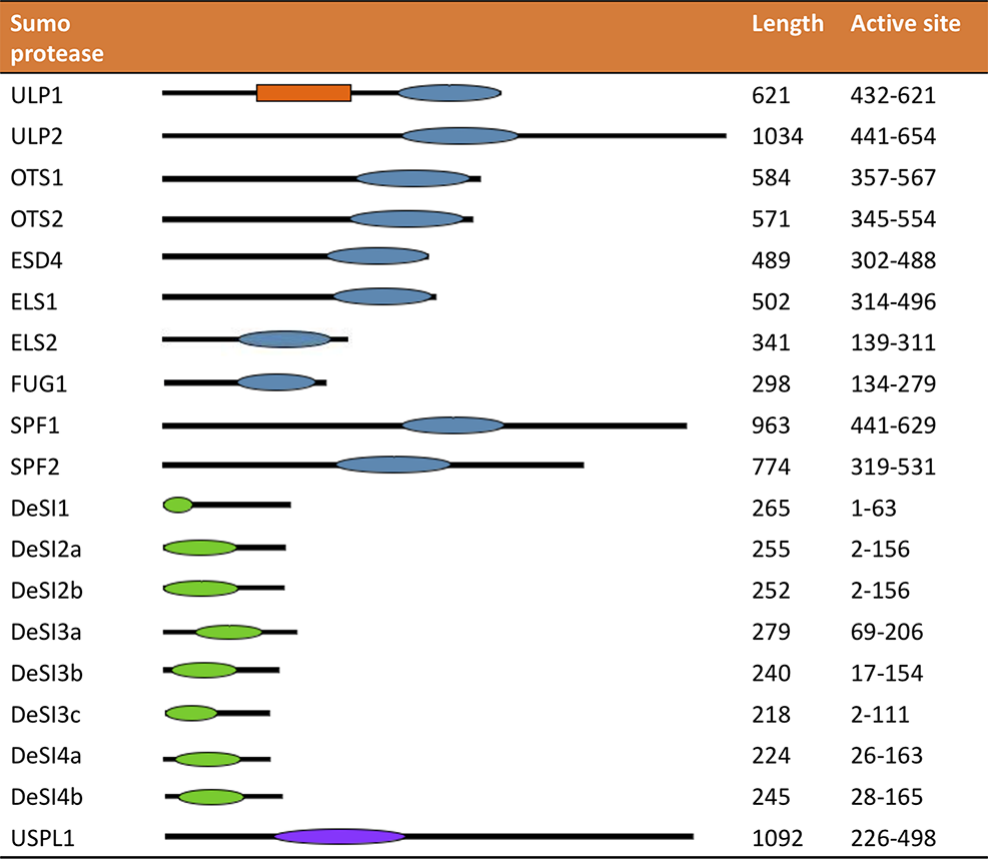
The location of the active site and length of several different SUMO proteases. The orange box denotes the SIM site in ULP1. The blue oval depicts the location of the C48 active site, the green oval the C97 active site, and the purple oval the C98 active site.

The domains and structures of plant SUMO proteases have not been well studied; they are largely based on similarity to yeast SUMO proteases. The ULP proteases typically have a 200–amino acid catalytic domain at the C-terminus of the protein (see **Figure 4**). The N-terminus of the protein is typically highly variable and is presumed to be responsible for specificity of SUMO protein–conjugate recognition and modulation of enzymatic activity and directing subcellular localization (Li and Hochstrasser, 2003; Gong and Yeh, 2006; Mukhopadhyay et al., 2006; Kroetz et al., 2009). The N terminal domain is also thought to contain SIM (SUMO-interacting motif) sites which may increase enzyme affinity for SUMOylated substrates; alternatively, they may help aid orientation of the SUMOylated proteins in the catalytic site (Hickey et al., 2012).

However, as can be seen in **Figure 4**, the structure of the ULP proteases varies between the proteases, helping to provide their individual specificity. The catalytic domain in ELS2 is closer to the N-terminus, and the catalytic domains in SPF1 and SPF2 are located in the center of the protein and form much larger proteins than the other ULP proteases. Chosed et al. (2006) examined the effect of truncating the OTS1, OTS2, ELS1, and ESD4, expressing just the catalytic domain *in vitro*. Truncated ESL1 and ESD4 were unable to function as SUMO proteases requiring the full length of the gene. Conversely, the truncated form of OTS2 was able to cleave more forms of SUMO than when the N-terminus of the protein was intact; the truncated form of OTS2 was able to cleave yeast and mammalian and tomato SUMO; this activity was not present in the full length of OTS2 (Chosed et al., 2006).

Additionally, the *Arabidopsis* ULP proteases have not had the crystal structure solved. It is assumed, however, that the structure will be similar to yeast ULP1. The crystal structure of yeast ULP1 catalytic domain interacting with SUMO has revealed a tight but shallow VDW tunnel that recognizes the Gly–Gly motif, stably orienting substrates to come into close interaction with the active site. The structure showed that side chains of residues other than glycine would sterically clash with the narrow tunnel, providing specificity (Mossessova and Lima, 2000). When the substrate is positioned in the VDW tunnel, the scissile bond after the double glycine is converted from a trans to a cis bond causing a kink in the SUMO C-terminal tail (Shen et al., 2006). This configuration is thought to be rare in proteins and is induced to destabilize the bond and promote cleavage (Hickey et al., 2012). The active site in ULP1 is in a narrow cleft structured to enable both large SUMO conjugates and single-SUMO molecules to access it (Mossessova and Lima, 2000). The active site is between two structural subdomains, one subdomain is a beta-barrel carrying the active site histidine and glutamine (or aspartic acid), and the second subdomain consists of a helical bundle, with one helix carrying the catalytic cysteine.

Compared to the ULPs, the DeSI proteins are smaller proteins with a larger component of their size comprising the active site (**Figure 4**), which is around 140 amino acids; the active site is also closer to the N terminus. As with the ULP proteases, the DeSI proteases have putative SIM sites (Hickey et al., 2012). The crystal structure for the DeSI proteases in *Arabidopsis* has not been solved but can be assumed to be similar to the solved structure of human DeSI1. It revealed the protein forms as a homodimer forming a papain groove between the two subunits forming the active site with the catalytic dyad. In the DeSI protein, the proteolytic groove forms between the cysteine at the N-terminal end of a helix, and the histidine on a strand that is part of a beta-barrel. Surprisingly, the C-terminal tails of DeSI1 were shown to fold into the groove, seemingly blocking access to the active site. However, activity assays with a truncated C-terminal tail show no effect compared to WT, suggesting the structure may be an effect of crystallization (Suh et al., 2012).

The domains and structure of USPL1 have not currently been characterized but can be hypothesized to have similar functions to that of the ULP proteases containing SIM site signals for cellular localization and providing substrate specificity.

## Evolution and Diversification of SUMO Proteases in Plants

**Figure 5:**
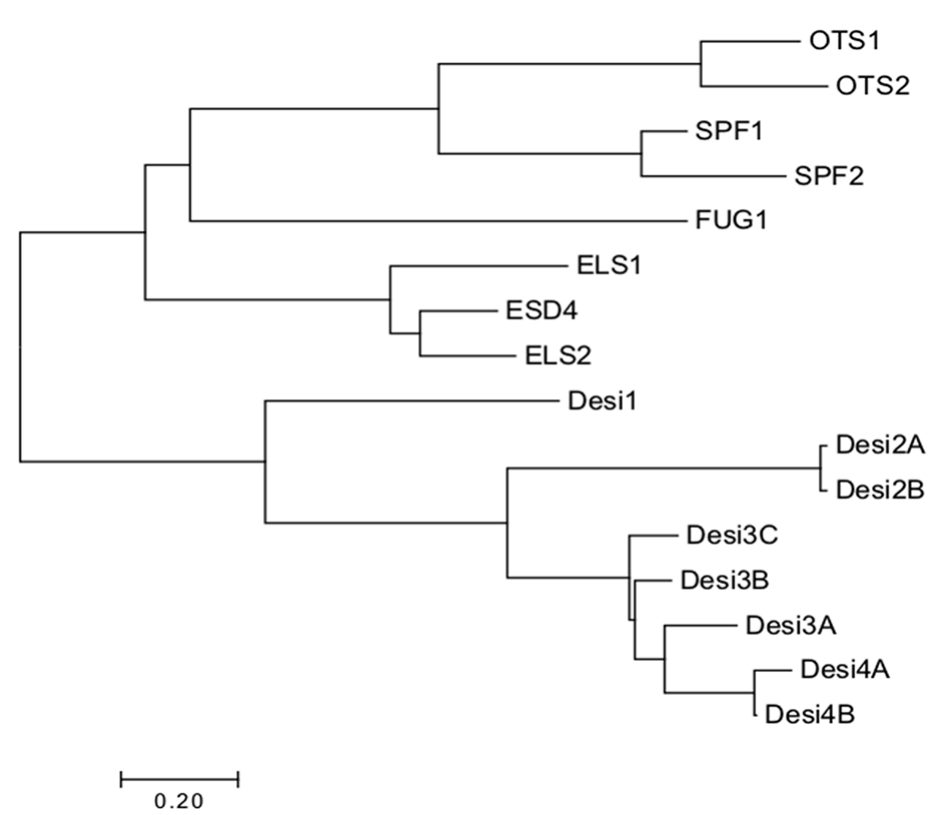
Phylogenetic tree of currently identified SUMO proteases in *Arabidopsis*. The proteases cluster according to their catalytic triad. Alignments were made using ClustalX and visualized in Jalview. Bootstrap neighbor-Joining trees were made using ClustalX and visualized using MEGA7.

The ULP SUMO proteases are evolutionarily distinct from the DeSI SUMO proteases (**Figure 5**). The ULP SUMO proteases have a phylogenetic origin that can be traced to green algae and other eukaryotes including yeast ULP1 and ULP2 (Castro et al., 2018a). The evolutionary classification of the proteases has proved difficult due to the high amino acid sequence divergence. Initially, OTS1 and OTS2 were classified in a group that was more closely related to ESD4/ELS1/ELS2 believed to be related to *Sc*ULP1 due to the active site being located at the C-terminus of the protein like *Sc*ULP1 (Novatchkova et al., 2004; Lois, 2010). However, based on amino acid conservation, they are more similar to *Sc*ULP2 (Castro et al., 2016). The current classification in use was carried out by Novatchkova et al. (2012), who conducted an in-depth phylogenetic analysis, including *Arabidopsis*, tomato, grapevine, and poplar genomes. They generated a novel grouping of the ULP proteases into four groups in *Arabidopsis*, namely, A, B1, B2, and C (Novatchkova et al., 2012). Benlloch and Lois (2018) suggested using the same classification system classing the ULP proteases on sequence similarity and organization of the ULP domain; however, they suggested classing the proteases independently of their similarity to yeast ULPs. Conversely, Castro et al. (2018a) used the classification system to divide the ULP proteases into evolution from ScULP1 and ScULP2; however, the naming system was changed and is being used in this review. Class I ELS type of homologues is believed to have evolved from ScULP1 including ESD4, ELS1, and ELS2. Three different subdivisions have evolved from ScULP2 including class II OTS-type homologues including OTS1 and OTS2 class III SPF-type homologues, including SPF1 and SPF2 and class IV FUG type that is yet to be characterized as a SUMO protease. However, the relationship of FUG1 to the other SUMO protease groups strongly suggests that it has the same activity (Novatchkova et al., 2012). FUG1 may be a relatively newer protease as it appears to be absent from early plant taxa, being present in flowering plants (Castro et al., 2018a).

The DeSI proteins are not as well studied evolutionarily due to their very recent identification in *Arabidopsis*; however, due to the similarities in some of their sequences, it can be hypothesized that they may share functional redundancy (Orosa et al., 2018), but this is yet to be determined.

If USPL1 homologues are found in *Arabidopsis*, it is likely that they will share more evolutionary similarity with the ULP proteases as clan CE proteases are hypothesized to share sequence similarity with clan CA proteases.

## Localization of SUMO Proteases in the Cell and Plant Tissue

**Figure 6:**
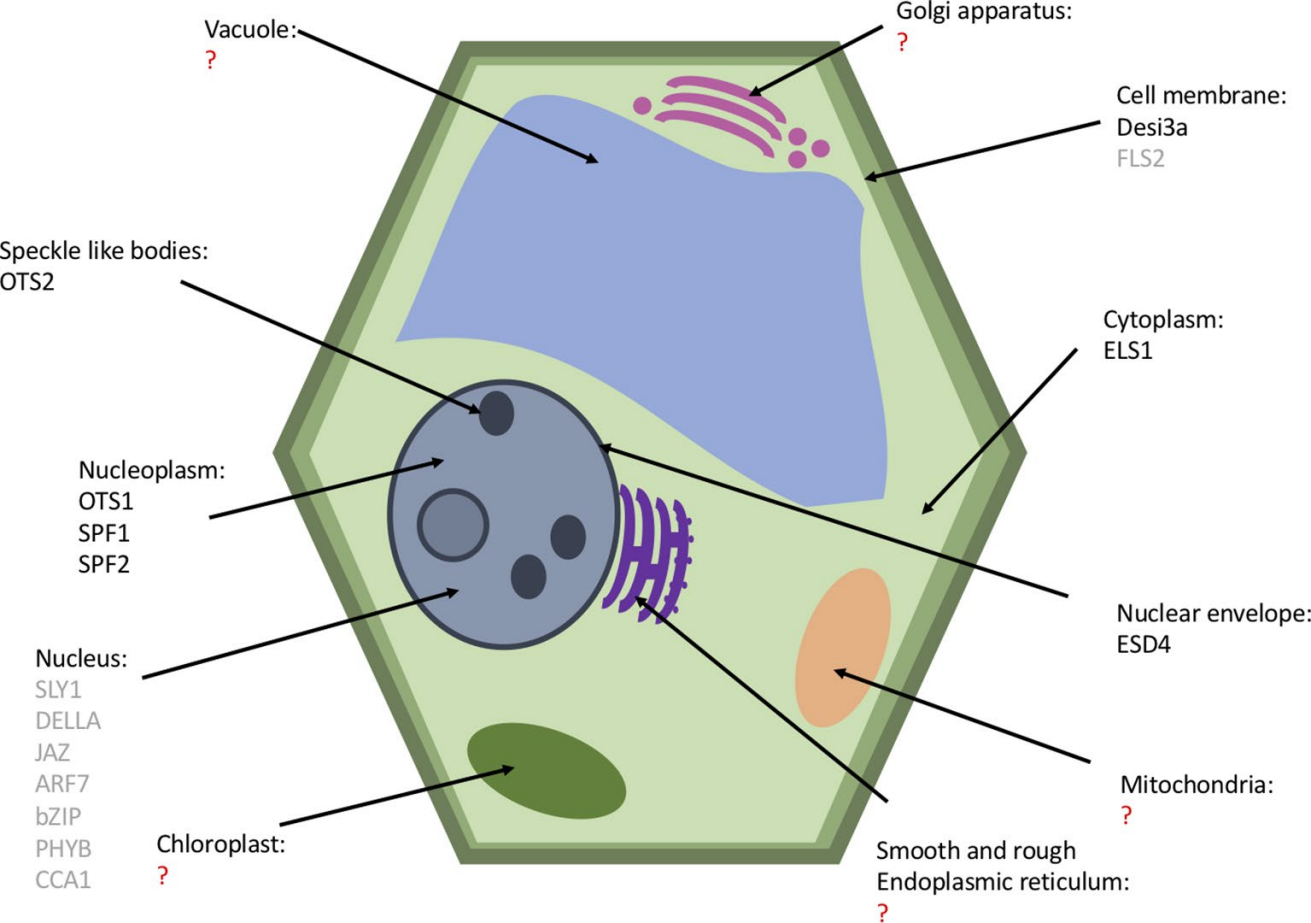
A plant cell featuring the known cellular location of SUMO proteases (black) and their substrates (gray). Organelles with a question mark (red) have no known SUMO proteases or substrates but may have undiscovered SUMO proteases.

The subcellular localization of the SUMO proteases is thought to provide specificity to the SUMOylation machinery (Chosed et al., 2006). Li and Hochstrasser (2003) expressed in yeast cells lacking *Sc*ULP2 a truncated form of *Sc*ULP1 with just the catalytic domain expressed; this mutant was capable of suppressing defects of cells lacking ULP2, whereas full length *Sc*ULP1 was unable to. This suggests that the N-terminal region of ULP1 restricted activity of the protease; this may have been its cellular location to enable proteolytic activity. The known subcellular localization of the *Arabidopsis* SUMO proteases are summarized in **Figure 6**. The cellular localization is largely based on the N-terminal sequence; deletion of the localization domain alters the targets that are deSUMOylated (Li and Hochstrasser, 2003; Gong and Yeh, 2006; Mukhopadhyay et al., 2006; Kroetz et al., 2009). Yeast ULPs all localize to the nucleus, *Sc*ULP1 localizes to the nuclear pore complexes in the nuclear envelope (Panse et al., 2003), and *Sc*ULP2 localizes to the nucleoplasm (Li and Hochstrasser, 2000). The ULP proteases largely all localize to the nucleus; ESD4 predominantly localizes to the periphery of the nucleus at the nuclear periphery and envelope (Murtas et al., 2003; Xu et al., 2007). Surprisingly, unlike the other ULP proteases, the closest homologue of ESD4, ELS1, is present in the cytosol (Hermkes et al., 2011). OTS1 and OTS2 localize to the nucleus, and OST2 is also found in nuclear foci (Conti et al., 2008). Additionally, the recent characterization of SPF1 and SPF2 showed that they are nuclear proteases (Kong et al., 2017; Liu et al., 2017a).

The DeSI1 proteases identified in human cells have been found to be located at both inside and outside the nucleus, localizing predominately in the cytoplasm, whereas DeSI2 localizes exclusively in the cytoplasm (Shin et al., 2012, Suh et al., 2012). The only DeSI currently characterized in *Arabidopsis*; DeSI3a was found to localize in the cell membrane by expressing mCherry-DeSI3a in *Nicotiana benthamiana*. Ultracentrifugation which separated the cytoplasmic nuclear and membrane fractions identified DeSI3a in the membrane fraction (Orosa et al., 2018).

The SUMO proteases are localized in all tissues in the plant; generally, their localization correlates with their phenotypes when knocked out. The highest concentration of ESD4 mRNA was detected in the inflorescence and flowers; however, it was also detected at lower concentrations in the seedlings, leaves, shoots, and roots of wild-type plants and was found to be constantly present throughout the 24-hour cycle (Murtas et al., 2003). An ELS1 promoter–GUS fusion showed that ELS1 is expressed ubiquitously in the plant with a higher accumulation in the vasculature and roots. RT-PCR demonstrated that there is also high expression in flowers and low levels of expression in the siliques and leaves (Hermkes et al., 2011).

Both OTS1 and OTS2 have a similar expression pattern as observed through GUS staining, both present from the early developmental stages, with high expression levels in vascular tissue in the root and shoot of seedlings and in the petioles. In mature plants, expression in the leaves was reduced, compared to the seedlings. The proteases were also identified in the flowers and siliques with OTS2 expression stronger than OTS1; for most other tissues, OTS1 expression was stronger (Castro et al., 2016).

The GUS reporter system for SPF1 expression found ubiquitous expression in 2-day-old seedlings; 4-day-old SPF1 was detected in the hypocotyl, cotyledons, and shoot and root apices of the seedlings. In older seedlings, it is present in newly developing leaves, shoot apex and root tips (Kong et al., 2017; Liu et al., 2017a). SPF1 and SPF2 strongest gene activity was detected in the reproductive organs, specifically localizing to embryo sacs, inflorescences, anthers, and developing seeds (Kong et al., 2017; Liu et al., 2017a). Despite SPF1 and SPF2 being expressed in the same tissues, both have different expression patterns in the respective tissues. Tissue-specific PCR revealed that SPF1 transcription is highest in inflorescences and cauline leaves, with intermediate expression levels in stems and rosette leaves. SPF2 transcription was seen to be at its highest in stems, cauline leaves, rosette leaves, and middle-length siliques, and interestingly, no expression of SPF2 was detected in root tissue (Liu et al., 2017a).

## SUMO Protease Specificity

The SUMO proteases provide specificity in the SUMO targets they cleave. This can include specificity in the SUMO isoform they cleave, whether they mature the SUMO isoform, and the target protein they cleave SUMO from. The specificity the SUMO proteases exhibit is summarized in **Table 2**.

**Table 2:** Summary of known specificity of SUMO proteases against the different SUMO isoforms in *Arabidopsis*. SUMO 5 is not included in the table as there is no currently identified protease for SUMO 5. M, matures SUMO; D, deconjugates SUMO; NT, not tested.

	SUMO 1		SUMO 2		SUMO 3	
	M	D	M	D	M	D
ESD4	High	High	High	High	–	–
ELS1	High	High	High	High	Low	–
OTS1	High	High	High	High	–	–
OTS2	High	High	High	High	–	–
SPF1	Medium	Low	–	NT	–	NT
SPF2	Medium	NT	–	NT	–	NT
Desi3a	NT	High	NT	NT	NT	NT

ELS1 is hypothesized to be more likely to be involved in SUMO maturation than in deconjugation. This is due in part to the observations that the loss of yeast ULP1 (the hypothesized SUMO maturase in yeast) can be rescued through ELS1 expression, and that *Arabidopsis els1-1* mutants show only slightly increased accumulation of high molecular weight SUMO conjugates, suggesting a limited role in SUMO regulation (Hermkes et al., 2011). Chosed et al. (2006) also found ELS1 to have greater SUMO peptidase than isopeptidase activity. ELS1 was shown to cleave SUMO1, 2, and 3 to generate mature SUMO. They also demonstrated that ELS1 can deconjugate SUMO1 and SUMO2 from target proteins; however, these studies were carried out *in vitro* (Chosed et al., 2006).

Surprisingly, given the high sequence similarity between ELS1 and ESD4, ESD4 was not capable of suppressing the yeast *ulp1* phenotype. In contrast, ESD4 was capable of complementing the *ulp2* yeast mutant temperature sensitivity. Yeast ULP2 predominantly cleaves polymeric SUMO chains on target proteins (Li and Hochstrasser, 1999; Schwienhorst et al., 2000; Bylebyl et al., 2003; Hermkes et al., 2011). This suggests that ESD4 may play a role in SUMO chain editing. ESD4 is capable of deconjugating SUMO1 and SUMO2 from target proteins, but not SUMO3. ESD4 also displays SUMO endopeptidase activity toward SUMO1 and SUMO2, but not SUMO3 (Chosed et al., 2006; Colby et al., 2006).

SPF1 has the weakest capabilities of cleaving SUMO1 from target proteins (Kong et al., 2017). Both SPF1 and SPF2 have SUMO peptidase activity toward SUMO1 but are unable to mature SUMO2 or SUMO3 (Liu et al., 2017a). SPF1 and SPF2 have SUMO deconjugase activity particularly in inflorescences; in *Arabidopsis spf-1* and *spf1-1 spf2-1* mutants, there was a greater number of higher molecular weight SUMO conjugates in inflorescences compared to WT and compared to seedlings (Liu et al., 2017a).

OTS1 and OTS2 are capable of deconjugating SUMO1 and SUMO2 from target proteins, but not SUMO3. OTS1 and OTS2 also display SUMO endopeptidase activity toward SUMO1 and SUMO2, but not SUMO3 (Colby et al., 2006; Conti et al., 2008).

In mouse, the DeSI proteins do not possess SUMO maturation activity; so far, they have only been involved with deconjugating SUMO and chain editing (Mukhopadhyay and Dasso, 2007; Shin et al., 2012; Suh et al., 2012). DeSI3a, the only currently characterized DeSI in plants was shown to cleave isopeptide-linked poly-SUMO chains (Orosa et al., 2018); however, SUMO peptidase activity was not tested.

## Physiological Effect of SUMO Proteases

**Figure 7:**
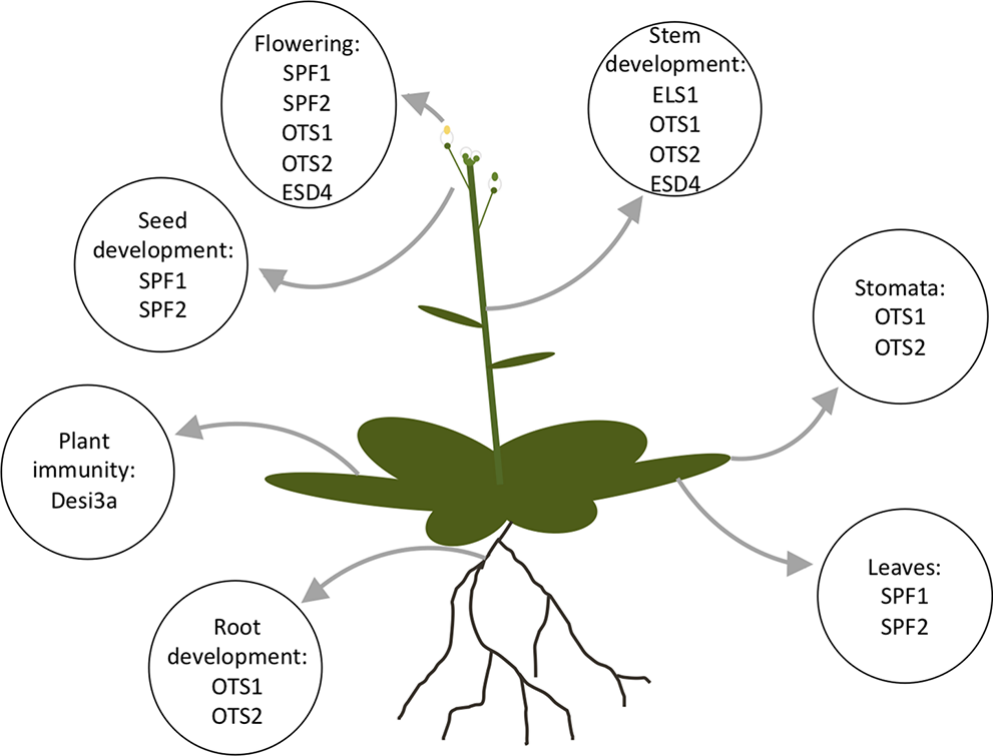
An *Arabidopsis* plant with labeled organs listing the proteases that are known to be present in the organ or effect the organ development.

SUMOylation has pleiotropic effects on cell dynamics, and currently over 1,000 proteins have been identified as SUMO targets (See Elrouby and Coupland, 2010; Miller et al., 2010; Rytz et al., 2018). Due to the importance of the SUMO proteases in regulating the SUMOylation in plants, alterations in SUMO protease expression levels can alter the development and physiology of the plant. The different physiological phenotypes of the SUMO proteases can provide an insight into the role of the individual SUMO proteases and their targets. The physiological phenotypes the SUMO proteases alter is summarized in **Figure 7**.

ESD4 was initially phenotyped by Reeves et al. (2002); Murtas et al. (2003) identified ESD4 as a SUMO protease. Reeves et al. (2002) identified the *esd4-1* mutant as having an early flowering phenotype, which was most obvious under short photoperiods. Additional phenotypes observed of the *esd4-1* mutant included premature termination of the shoot and an alteration of phyllotaxy along the stem, giving a dwarf stature and deformed siliques, irregularly positioned along the stem (Reeves et al., 2002). The early flowering was determined to be in part due to the level of the floral repressor FLC mRNA being reduced in *esd4-1* mutants; however, it was also suggested that ESD4 also promotes flowering independently of FLC (Reeves et al., 2002). Furthermore, the phenotype was enhanced when SUMO1, 2, and 3 were overexpressed in the *esd4-1* mutant (Murtas et al., 2003).

*Els1-1* mutants appear similar to wild type except for slightly reduced growth and thinner stems (Hermkes et al., 2011). *Els1-1* knockout mutants were analyzed for difference in flowering time, due to ELS1 close homologue ESD4 having such an obvious phenotype; however, *els1-1* mutants showed no strong statistically significant difference between WT and *els1-1* (Hermkes et al., 2011).

The SPF single and double mutants do not display a severe phenotype in early stages of development (Castro et al., 2018b). At the flowering stage, however, the *spf1-1* mutant has a late flowering phenotype, which is more pronounced under long days (Kong et al., 2017). *Spf2-1* mutants exhibit no clear phenotype, and the *spf1-1 spf2-1* double mutant has a greater flowering delay than the single mutants (Castro et al., 2018b). In addition to the timing of flowering, *spf1-1* has shorter siliques and abnormal seeds; this was not observed in the *spf2-1* mutants (Liu et al., 2017a). In the *spf1-1 spf2-1* double mutants, overall seed production was reduced with the mutants producing larger seeds (Liu et al., 2017a; Castro et al., 2018b). As has been observed with the other phenotypes, *spf2-1* did not show a difference in flowers compared to WT; however, *spf1-1* and *spf1-1 spf2-1* mutants had two thirds of flowers with abnormally long styles, which causes a physical fertility barrier (Liu et al., 2017a). Additionally, the *spf1-1* and *spf1-1 spf2-1* mutant flowers had fewer pollen grains. The reduced fertility in the *spf1-1* and *spf1-1 spf2-1* mutants was partially due to abnormal microgametophytes. Many pollen grains were shown to be non-viable with abnormal shapes and reduced cytoplasmic content, implicating SPF1 and SPF2 in pollen grain development (Liu et al., 2017a). Ovule development was also analyzed; abnormal ovules were only observed in the *spf1-1 spf2-1* double mutant, showing arrested embryo sacs and degeneration of embryo sacs which may be due to the regulation of callose degradation (Liu et al., 2017a). Particularly in the *spf1-1 spf2-1* double mutant, abnormal embryos were detected with irregular morphologies (Liu et al., 2017a). The physiological observations of abnormal fertility organs were further supported genetically as fertility gene expression was also abnormal in the *spf1-1* and *spf1-1 spf2-1* mutant plants compared to WT. Finally, *spf1-1* and *spf1-1 spf2-1* mutants displayed altered leaf morphology showing elongated darker leaves due to an accumulation of chlorophyll, carotenoids, and anthocyanins (Castro et al., 2018b). Microarray analysis demonstrated that differentially expressed genes were involved in cell wall and secondary metabolism including genes pertaining biosynthesis of phenylpropanoids, glucosinolates, and lipids (Castro et al., 2018b).

Double mutants of *ots1-1 ots2-1* have a small stature and early onset flowering; this phenotype was not observed in the single mutants. Additionally, the *ots1-1 ots2-1* double mutant, but not the single mutant, show reduced shoot weight, rosette radius, number of leaves, and late germination (Castro et al., 2016). The observed developmental defects were further supported by microarray analysis of *ots1-1 ots2-1* showing overrepresentation of genes related to shoot development including organ morphogenesis (Castro et al., 2016). The *ots1-1 ots2-1* double mutants also produce a lower number of seeds and have a significantly reduced filament length. Some of the observed phenotypes in *ots1-1 ots2-1* double mutant are reversed to WT conditions if either a DELLA protein is also knocked out from the mutant such as RGA (repressor of ga1-3) or GAI (gibberellin-insensitive), or if GA (gibberellins) or JA (jasmonic acid) is applied (Campananaro et al., 2016). This suggests that the fertility phenotype of *ots1-1 ots2-1* is controlled through the DELLA proteins (Campananaro et al., 2016). It has been demonstrated that the DELLA proteins are SUMOylated, with SUMO stabilizing the DELLA proteins and OTS1/2 are the proteases that cleave SUMO from the DELLAs (Conti et al., 2014). In the *ots1-1 ots2-1* double mutant, there are increased levels of DELLAs, due to the SUMOylated DELLAs being stabilized (Conti et al., 2014). The higher accumulation of DELLAs in the *ots1-1 ots2-1* mutant results in reduced fertility in the plants; this phenotype is reversed in a triple mutant of *ots1-1 ots2-1 della* mutant (Campananaro et al., 2016). Finally, *ots1-1 ots2-1* double mutant also has an increased stomatal aperture compared to WT (Castro et al., 2016).

The knockout mutant of *desi3a* does not have differences in global SUMOylation immunoblots, compared to WT. Additionally, no obvious physiological phenotype was reported in Orosa et al., 2018; it was hypothesized due to DeSI3a having a narrow range of targets (Orosa et al., 2018).

## Role of SUMO Proteases in Plant Hormonal Pathways

SUMO may provide a key point of cross talk between the different PTMs as SUMOylation can act as a signal for ubiquitination of proteins (Elrouby et al., 2013) and can also regulate kinases and phosphatases (Crozet et al., 2014). This enables SUMO to act as a central regulator of signaling and enables the PTMs to coordinate complex molecular responses (Garrido et al., 2018). This may enable the SUMO system to exert control over hormonal responses in plants. **Table 3** summarizes the currently identified SUMO proteases that have been shown to have roles in the hormonal pathways.

**Table 3:** Table of known hormones in plants and identified SUMO proteases that have a role in the hormone pathway, highlighting the hormone pathways where SUMO proteases have not currently been identified to have a role, which may be a research opportunity.

Hormone	Role of hormone	SUMO protease
Gibberellin	Plant growth, floral development, fruit growth	OTS1, OTS2, ESD4
Auxin	Apical dominance, tropism, branching, lateral roots	
Cytokinin	Releases lateral buds from apical dominance, delays senescence	
Ethylene	Flowering/fruit ripening, stress response, seed germination	
Abscisic acid	Stomatal closure, drought response, seed maturation, germination, root shoot growth	OTS1, OTS2, SPF1, SPF2
Jasmonic acid	Plant defense from insects, necrotrophy pathogen response, root growth	OTS1, OTS2
Salicylic acid	System acquired resistance to pathogens, biotrophic pathogen	OTS1, OTS2, ESD4
Brassinosteriods	Cell division/elongation in stem/roots, photomorphogenesis, reproductive development, leaf senescence	OTS1, OTS2
Strigolactone	Branching, leaf senescence, root development, plant microbe interaction	

*Esd4-1* mutants accumulate elevated levels of SA (salicylic acid). The *esd4-1* phenotype is partially alleviated by mutation of salicylic acid biosynthesis gene ICS1 (isochorisate synthase1). Double *esd4-1 ics1-1* mutants are larger and flower later than *esd4-1* mutants and accumulate less SA. They also accumulate fewer SUMO conjugates, with levels falling back to wild type or slightly above wild type depending on the background (Villajuana-Bonequi et al., 2014). This last observation implies that the increase in SUMO conjugates visible in *esd4-1* mutants may be caused in part by an increase in SUMOylation rather than by a decrease in deSUMOylation. While, the inactivation of ICS1 reduces the levels of SUMO conjugates in *esd4-1* mutants, it does not increase the levels of free SUMO1/2 (Villajuana-Bonequi et al., 2014) as would be expected in the case of increased deconjugation. However, the exact relationship between SA and free SUMO is still unknown. A variety of SUMO-related mutants, including *esd4-1*, *ots1-1 ots2-1*, *siz1-1*, and *sum1-amiR sum2*, and SUMO overexpression lines show hallmarks of an increased SA response (van den Burg et al., 2010; Villajuana-Bonequi et al., 2014; Bailey et al., 2016).

Microarray analysis of *spf1-1 spf2-1* observed upregulation of genes associated with auxin, brassinosteroid, cytokinin, gibberellin, jasmonate, and salicylic acid hormones. It was observed that *spf1-1* and *spf2-2* mutants are less sensitive to ABA (abscisic acid) treatment; the induction of ABA response genes was reduced in *spf1-1* mutant plants compared to WT. It was demonstrated that SPF1 is capable of regulating ABI5 (ABA-insensitive5) and MYB30 (MYB domain protein30); these proteins regulate ABA signaling during early seedling development. In *spf1-1*, mutant plants ABI5 and MYB30 accumulate to greater levels than in WT. SPF1 transcription increases in ABA treated seeds, but not in seedlings (Wang et al., 2018).

OTS1/2 also shows an ABA phenotype, *ots1-1 ots2-1* double mutants have a lower germination success rate, and this physiological trait can be a marker of ABA. The root length of *ots1-1 ots2-1* is shorter on ABA, compared to WT, and germination is delayed in *ots1-1 ots2-1* grown on ABA. Additionally, the stomatal size of *ots1-1 ots2-1* is greater than WT when ABA is added (Castro et al., 2016).

The double mutant *ots1-1 ots2-1* is less sensitive to JA (jasmonic acid), than WT (Srivastava et al., 2018). This is believed to be due to JAZ6 (jasmonate-ZIM-domain protein6) and JAZ1 (jasmonate-ZIM-domain protein1), JA repressor proteins, being SUMOylated and deSUMOlyated by OTS1/2. SUMOylation of the JAZ repressors stabilizes the proteins; SUMOylated JAZ6 is less capable of interacting with the JA receptor COI1 (coronatine-insensitive1). Stimulated by JA, COI1 binds to JAZ6 mediating the degradation of JAZ6 *via* the ubiquitin pathway. The *ots1-1 ots2-1* mutants display JA insensitivity as the JAZ proteins are more SUMOylated in the *ots1-1 ots2-1* background and thus are more stable; due to less interaction with COI1, the JAZ6 repressors block downstream signaling of JA (Srivastava et al., 2018).

OTS1/2 have also been shown to play a role in the SA pathway. The *ots1-1 ots2-1* double mutant has more SA signaling genes upregulated. This may be due to SA biosynthesis genes also being upregulated in the *ots1-1 ots2-1* mutant including ICS1, an SA biosynthesis gene, which is further increased when the plants are subjected to SA or an SA functional analogue. The mutants may lack restrictive regulation of ICS1 gene transcription. Furthermore, OTS1/2 are degraded in SA suggesting that they negatively regulate SA signaling and provide a feedback mechanism. In high levels of SA, OTS1 and OTS2 are degraded; when OTS1 and OTS2 are more abundant, they lower SA levels by reducing ICS1 levels, and this may be *via* deSUMOylation of a transcription factor that has not yet been identified (Bailey et al., 2016).

SUMOylation also determines the interaction of GA (gibberellins) receptors with DELLA proteins. GA is a growth promoting hormone, stimulating the degradation of growth-repressor DELLA proteins. SUMOylated DELLA proteins are stabilized and therefore accumulate and act to inhibit growth through DNA binding. DeSUMOylation of DELLA proteins by OTS1 allows these proteins to interact with the GA receptors GID1 (gibberellin-insensitive dwarf1), resulting in their degradation and therefore repression of the DELLA inhibitory pathway, thus allowing plant growth. The double mutant *ots1-1 ots2-1* has increased levels of DELLA due to higher levels of SUMOylation and therefore stabilization of the protein. When SUMO is conjugated to DELLA, it changes the conformation of GID1 (the GA receptor that promotes degradation of DELLA *via* the ubiquitin pathway) through a SIM site in GID1, preventing GID1 from promoting degradation of DELLA (Conti et al., 2014).

Most studies of the posttranslational control of the GA pathway focus on the DELLA proteins, the repressor proteins of the hormonal pathway. The DELLA proteins interact with and control the stability of SLY1 (sleepy1) which forms the Skp, CULLIN, F-box (SCF), and complex of E3 ubiquitin ligases that polyubiquitinate and degrades the DELLA proteins. SLY1 encodes an F-box protein that provides substrate specificity of the SCF complex recognizing and binding the DELLA proteins. SLY1 is SUMOylated by SIZ1, a SUMO E3 ligase, upon SUMOylation SLY1 is stabilized and activated. A non-SUMOylatable mutated SLY1 protein has the same dwarf phenotype as a *sly1-1* knockout plants. The SUMO protease ESD4 was identified as the SUMO protease cleaves SUMO from SLY1. In *esd4-1* mutant plants, there were higher levels of SLY1 protein, due to higher levels of SLY1 SUMOylation resulting in stability and activation of SLY1, degrading more DELLA proteins resulting in more GA signaling (Kim et al., 2015).

## Responses to Biotic Environment

The role of SUMO proteases in immunity has been speculated for some time as plant pathogens including *Xanthomonas campestris*, *Ralstonia solanacearum*, *Pseudomonas syringae*, *Erwinia pyrifoliae*, and *Rhizobium* spp. utilize effector proteins, which are injected into plants to overcome the host defense, which have sequence homology to ULP SUMO proteases and display efficient isopeptidase activity to SUMO1/2 and SUMO3 (Orth et al., 1999; Orth et al., 2000; Deslandes et al., 2003; Hotson et al., 2003; Hotson and Mudgett, 2004; Roden et al., 2004; Bartetzko et al., 2009; Kim et al., 2013). The bacteria that causes bacterial spot (*X. campestris* pv. *vesicatoria* [*X.c.v.*]) does not possess an endogenous SUMOylation system; however, it injects an effector into host cells that is capable of deSUMOylating protein targets in host cells and prevent hypersensitive response (Orth et al., 2000). This highlights the important role of SUMO proteases in plant immunity and how modulating SUMO protease activity can play a critical role in pathogen resistance. Understanding the SUMOylated proteins the effector proteases are targeting may help provide information on important SUMOylated substrates in plant pathogen defense.

DeSI3a is a cell membrane–bound SUMO protease that plays a role in PAMP (pathogen-associated molecular patterns) detection. Upon detection of flagellin, FLS2 is SUMOylated triggering the release of BIK1 (Botrytis-induced kinase1), a cytoplasmic kinase resulting in downstream signaling in innate immunity. When flagellin is perceived, DeSI3a is degraded which enhances FLS2 SUMOylation, triggering BIK1 dissociation and downstream intracellular immune signaling. Mutant *desi3a-1* plants do not have different global SUMO levels compared to WT, suggesting that DeSI3a acts on a narrow range of targets; however, it did exhibit increased transcript levels of key defense genes upon treatment with flg22 (flagellin22). Additionally, FLS2 in the *desi3a-1* background exhibited hyper SUMOylation compared to FLS2 in the WT background, this in turn resulted in a greater release of BIK1 in the *desi3a-1* background due to greater ROS (reactive oxygen species) burst levels and MAPK (mitogen-activated protein kinases) activation in this background (Orosa et al., 2018).

OTS1 and OTS2 have been implicated in having a role in pathogen defense; the *ots1-1 ots2-1* double mutant has increased resistance to *Pst* DC3000 (*P. syringae* pv. *tomato*). This may be due to the increased expression of PR1 and PR2 (pathogenesis-related 1/2) (pathogen defense genes) and higher levels of SA, caused by higher expression of SA biosynthesis genes such as ICS1. SA binds in plants to pathogen-related proteins that interact with transcription factors activating SA-mediated defense and enabling hypersensitive cell death response (Halim et al., 2006).

While *esd4-1* mutants have not been phenotyped for pathogen resistance, it can be hypothesized that it may have increased pathogen resistance. This is because the *esd4-1* mutant exhibit increased levels of SA which provides resistance to pathogens. Additionally, the *esd4-1* mutant has increased expression of PR1, a pathogen defense gene (Villajuana-Bonequi et al., 2014).

The *ots1-1 ots2-1* double mutant is more susceptible to *Botrytis cinerea* necrotrophs, having larger nectrophic lesions than WT likely due to *ots1-1 ots2-1* double mutants being less sensitive to JA. With some exceptions, JA usually activates defense against necrotrophic pathogens and herbivorous insects, whereas SA is often used in response to biotrophic pathogens (Glazebrook, 2005; Howe and Jander, 2008). As has already been described, in the *ots1-1 ots2-1* mutants, JAZ6 and JAZ1 are SUMOylated and stable. During *B. cinerea* infection, JAZ6 SUMOylation is enhanced and OTS1 degradation occurs. This results in *ots1-1 ots2-1* plants exhibiting more sensitivity to *B. cinerea* pathogens, as JAZ6 is more SUMOylated and is more stable and less able to interact with COI1, blocking the JA signaling cascade (Srivastava et al., 2018).

## Responses to Abiotic Environment

Due to the SUMO proteases playing a key role in responding to stress, alterations in SUMO protease protein levels may not provide a clear phenotype under healthy conditions. The phenotypes may only be observed when a plant is stressed.

OTS has been shown to influence many responses to the environment. Initially, OTS1 was identified due to high salt sensitivity; *ots1-1 ots2-1* root growth is significantly inhibited when grown on high salt. In high salt environments, OTS1/OTS2 is degraded while gene transcription remains unchanged (Conti et al., 2008). Similar to salt stress, OTS1/OTS2 have also been identified with having a role in osmotic stress. These two proteases have been identified as the proteases that removes SUMO from ARF7 (auxin response factor7), a transcription factor that induces expression of its target genes in an asymmetric manner in lateral root founder cells, providing roots with hydropatterning. When the root is in a wet environment, e.g., surface contact with an agar plate, ARF7 is deSUMOylated by OTS1/2 resulting in transcription of AF7 target genes and lateral roots formation in the direction of the water. When the root is in a dry environment e.g., the part of the root above the agar, ARF7 remains SUMOylated enabling interaction with IAA3 (indole-3-acetic acid inducible3), a repressor protein with a SIM site, preventing downstream transcription of ARF7 target genes. This prevents lateral roots growing in dry environments (Orosa-Puente et al., 2018). Placing *ots1-1 ots2-1* seedlings on media containing PEG (polyethylene glycol) or mannitol demonstrated that the double mutant is hypersensitive to osmotic stress, which may demonstrate that the proteases promote resistance to osmotic stress and not the ionic component of salt stress. This may be due to *ots1-1 ots2-1* having increased stomatal aperture (Castro et al., 2016). In rice, knocking out OTS1/2 promotes drought tolerance; rice OTS1-RNAi lines are much more sensitive to ABA and survive better in drought conditions losing less water. *Os*OTS1 interacts with *Os*bZIP (basic leucine zipper domain), a transcription factor that regulates ABA and drought responses. In the *Os*OTS1 RNAi lines, *Os*bZIP23 has higher levels of SUMOylation and is stabilized, leading to the transcription of more drought tolerant genes. *Os*OTS1 is degraded by exposure to desiccation, mannitol, and ABA, working as a feedback loop to stabilize *Os*bZIP23 under drought conditions (Srivastava et al., 2017). Srivastava et al. (2016a), also identified that rice *Os*OTS1 RNAi lines have a lower germination success rate; this physiological trait can be a marker of ABA (Srivastava et al., 2016a; Srivastava et al., 2016b). Castro (2013) reported a single mutant phenotype that *ots1-1* single mutants have an increased drought tolerance, while *ots2-1* mutants exhibit the same level of phenotypic drought tolerance as WT controls (Castro, 2013).

Another abiotic stress the OTS SUMO proteases have a role in is light. PHYB (phytochrome B) is a light absorbing photoreceptor that cycles between active and inactive states and switches to regulate photomorphogenesis. PHYB interacts with PIF (phytochrome-interacting factor) in low light levels which blocks photomorphogenesis. PHYB is SUMOylated in response to light; in low light levels, PHYB has low levels of SUMOylation; in high light, the levels of SUMOylated PHYB largely increase. When PHYB is SUMOylated, it blocks the interaction of PHYB with PIF5, inhibiting elongation and promoting growth. SUMOylation desensitizes PHYB signaling. OTS1/2 regulate PHYB action by deconjugating SUMO from PHYB. OTS1 therefore controls fundamental processes in plants such as inhibition of transcription factors and photomorphogenesis (Sadanandom et al., 2015).

OTS1 has also been implicated in copper tolerance; high levels of copper in the soil can cause plant toxicity. *Ots1-2* knockout mutant exhibited increased sensitivity to excess copper. Under excess copper, OTS1 regulates photosynthetic activity and ROS accumulation. When WT plants are subjected to a high dose of copper, the levels of SUMOylated proteins in a WT plant risesteadily, then fall with time. However, *ots1-2* plants have a constantly high level of SUMOylated proteins that does not show much of a response to the high levels of copper. OTS1 was shown to function in copper uptake and distribution; in the *ots1-2* mutant, more copper was detected in seedlings shoots and roots compared to WT; additionally, these knockout plants had greater expression of genes responding to high copper when placed on excess copper media than WT (Zhan et al., 2018).

Lastly, OTS1 and OTS2 also have a role in controlling the plants biological clock. CCA1 (circadian clock-associated1) is a novel regulator of key clock proteins and is SUMOylated at the end of the night/dawn phase. When CCA1 is SUMOylated, DNA binding affinity is reduced, which was seen in the *ots1-1 ots2-1* double mutant. The results demonstrate that SUMOylation also plays a role in regulating the biological clock in plants; CCA1 binds to over 1,500 gene promoters; imbalances in SUMOylation of CCA1 could have wide consequences for the growth and health of plants (Hansen et al., 2018).

## Future Research

Analyses of SUMO proteases using gain-of-function and loss-of-function studies have shown involvement in various cellular processes such as hormone signaling, plant defense, abiotic stress, enzyme activity, cell cycle progression, and plant development (Melchior, 2000; Lois et al., 2003; Murtas et al., 2003; Conti et al., 2008; Budhiraja et al., 2009; Hickey et al., 2012; Conti et al., 2014; Nelis et al., 2015; Sadanandom et al., 2015; Bailey et al., 2016). The SUMO proteases have been demonstrated to be important molecules in controlling these processes. However, there still remain some important questions that have yet to be answered on SUMO proteases which the last section will highlight.

### Unidentified SUMO Proteases

Currently, there are many SUMO proteases that have been identified through bioinformatic techniques but have not yet been functionally characterized using genetic, physiological, and biochemical approaches. These uncharacterized proteases include all but one of the DeSI proteases and two ULP proteases, FUG1 and ELS2, as have been earlier highlighted. Characterizing these proteases and understanding their molecular targets and localization may spread more light on the many pathways SUMO has a role in.

Despite this, however, among the SUMO machinery, the SUMO proteases are the most numerous family members, and they also show substrate specificity. Due to a small fraction of proteins being SUMOylated at a given time, SUMO proteases may play an important regulatory role in SUMOylation (Chosed et al., 2006; Colby et al., 2006; Yates et al., 2016; Benlloch and Lois, 2018). A large number of SUMO proteases may be required as SUMOylation specificity comes from spatiotemporal determinants, and thus, such a large number of proteins are required (Psakhye and Jentsch, 2012). Additionally, there is a large variation in the N-terminal domain of the SUMO proteases, which may provide specificity for the proteases. Some PTMs confer specificity by providing modifiers to protein complexes. In deubiquitinating enzymes (DUBs), additional levels of regulation are applied to the enzymes by forming protein complexes with “modifier” proteins. Scaffold proteins ensure DUBs with low affinity, but good catalytic capability for ubiquitin has substrates in close proximity. Adaptor proteins can bind to the DUB/scaffold protein and target protein to bring the ubiquitin into close proximity. These additional proteins ensure the correct protein substrate, and DUBs are in the right place at the right time; these types of proteins have not been identified in the SUMO system yet. Instead, the large variety in the N-terminal domains in the SUMO proteases may confer the role of these additional modifiers to the SUMO proteases, thus requiring a large number of differing SUMO proteases.

However, compared to the ubiquitin system, there is a much smaller number of SUMO proteases compared to ubiquitin proteases. To date, there are almost 100 different ubiquitin proteases belonging to five different protease families (Komander et al., 2009; Reyes-Turcu et al., 2009). It is likely that there are many other SUMO proteases to be identified. Some proteases that have currently, *via* bioinformatic techniques, been identified as a ubiquitin protease but have not yet been characterized may in fact be a SUMO protease, as was the case for USPL1 (Schulz et al., 2012).

Using predictive bioinformatic tools on already existing data may enable the identification of novel SUMO proteases. It is helpful that there are some known missing SUMO proteases, and this enables targeted searching. Some known missing SUMO proteases are proteases capable of maturing and deconjugating SUMO5, and deconjugating SUMO3 isoforms remain to be identified (Chosed et al., 2006; Colby et al., 2006). Furthermore, SUMO proteases may also play a role in additional modern techniques that could be utilized particularly to understand the proteases substrate specificity using molecular modeling and molecular dynamics methods. Additionally, it is likely that the SUMO proteases act in a complex web with some acting redundantly. Deciphering the role of individual SUMO proteases may require complex systems biology.

In addition to identifying more plant endogenous SUMO proteases, more research is also required into the pathogen effector molecules injected into plants that have SUMO protease activity. Plant pathogens infect plants with effector molecules that help dismantle host perception machinery and degrade host defense structures. As has earlier been discussed SUMO proteases have already been identified as playing a role in plant pathogen defense; effector molecules from various pathogens have been shown to have ULP protease homology and capable of deSUMOylation. There is a wide variety of pathogens and hosts, and each may have a different novel action of SUMO proteolytic activity. Additionally, understanding the SUMOylated substrates, the SUMO proteases target may help provide more information in pathogen disease progression and provide molecular targets to combat the disease. Currently of the identified pathogen effector molecules that have been identified most of the host target molecules are unknown.

In addition to SUMO proteases having a critical role in plant-pathogen interactions, the other major biotic stress plants can respond to is herbivorous attack. The role of SUMO proteases has not yet been explored. Due to the importance of SUMO proteases in responding to stress, it is likely that they will play a role in herbivore defense. A better understanding of these mechanisms may provide breeding targets in crop species to better protect crops against herbivore attack.

### Research in Crops

SUMO has been proven to have a critical role in stress responses (Kurepa et al., 2003; Miura et al., 2007; Saracco et al., 2007; Golebiowski et al., 2009), and it has been hypothesized that the SUMO proteases provide the SUMO system with specificity (Yates et al., 2016). This has led to the hypothesis that the crop species may have a greater number of SUMO proteases due to the selection pressures that have been applied to domesticated plants and bred in for stress tolerance (Augustine et al., 2016; Garrido et al., 2018). Generally, this has been observed in maize, with nine proteases identified based on sequence similarity to *Arabidopsis* ESD4 and OTS1 and 2 (Augustine et al., 2016). The crop *Brassica* species (maize, rice and sorghum) were all found to have many more sequences that matched ULP protease domain than four other members of non-domesticated *Brassicas* that were examined, suggesting that crop domestication may result in diversification of the SUMO proteases (Garrido et al., 2018).

However, there has, currently, been limited research into the SUMO system in crops. Many components of the SUMO system have been identified by bioinformatic techniques for rice, wheat, maize, and soybean (Yates et al., 2016; Li et al., 2017), and the maize SUMO system has been reconstituted (Augustine et al., 2016). Of the limited research carried out on crops, mainly knockout and overexpression lines of SUMO proteases, they have shown phenotypes demonstrating their importance in crops. Rice SUMO protease knockout lines with sequence homology to *Arabidopsis* ELS1 and FUG1 have been generated and examined with the plants showing dwarf phenotype, defects in fertility, seed weight, and flowering timing (Rosa et al., 2018). Additionally, rice knockout lines of *Os*OTS1 have shown reduced germination rate and reduced primary root growth (Srivastava et al., 2016a). RNAi rice lines of *Arabidopsis* OTS1 and OTS2 show salt hypersensitivity (Srivastava et al., 2016b; Srivastava et al., 2017), much like the *Arabidopsis* *ots1-1 ots2-1* double mutant (Conti et al., 2008). Furthermore, over expressing *Arabidopsis* OTS1 in wheat led to improved plant growth under water stress in addition to higher moisture content, photosynthesis rate, and chlorophyll content (le Roux et al., 2019).

From limited research into the crop SUMO proteases, they are already proving to be vital to crop stress survival. Further studies into a wider variety of crops will likely provide greater insights into their important role in stress survival and may prove useful targets for breeders.

### Regulation of Proteases

Understanding the regulation of the SUMO proteases can help explain how they are controlled and how they exert specificity to the SUMO system. The SUMO proteases are hypothesized in part to provide specificity by their specific expression, localization, regulation, sequestration, and degradation, although not many of these aspects are known for the SUMO proteases. Improving sophistication of cellular and molecular imaging may help shed light on how plants regulate the spatiotemporal location of the SUMO proteases.

A number of experiments have shown that the SUMO proteases are degraded by the ubiquitin protease system. During stress SUMO conjugates accumulate (Kurepa et al., 2003), which protects the plants, these conjugates may accumulate due to degradation of SUMO proteases by PTMs. OTS1 and OTS2 are degraded in high SA levels (Bailey et al., 2016), and DeSI3a is degraded by high flagellin levels (Orosa et al., 2018).

Transcription of the SUMO proteases may also react to stimulus—for example, SPF1 transcript levels increase in response to ABA (Wang et al., 2018). However, largely the transcriptional regulation and post translational modification of these proteins are not well understood. Elucidating the regulation of the proteases may help explain the role the proteases play in the SUMO system.

### Role of SUMO Protease in a Dynamic Cell

When analysis of the *Arabidopsis* SUMOylome was carried out, many SUMOylated targets were found to be present in the nucleus (Miller et al., 2013). Many proteins were known to be involved with histones, chromatin remodeling, transcription activators, co-repressors, and DNA repair (Miller et al., 2013), and substrates related to chromatin and RNA-dependent processes in *Arabidopsis* are SUMOylated (Budhiraja et al., 2009). These substrates are involved in processes that include regulation of chromatin structure, splicing, translation, DNA endoreduplication, and DNA repair. This has been supported by similar studies in animal and yeast cells (Psakhye and Jentsch, 2012; Seifert et al., 2015). As the vast collection of proteins SUMO targets involve DNA, RNA, and proteins that interact with these biomolecules such as transcription factors, coactivators and repressors, and chromatin modifiers, it suggests that stress-induced SUMOylation targets these components, potentially rewiring the chromatin and changing the transcriptional environment, enabling plant survival (Saracco et al., 2007; Miller et al., 2013; Augustine and Vierstra, 2018; Rytz et al., 2018). This is further supported by SUMO pathway loss-of-function mutants, in particular SUMO protease mutants, showing stress sensitive response in *Arabidopsis* and rice (Miura et al., 2007; Conti et al., 2008; Srivastava et al., 2016b; Srivastava et al., 2017). It is already known that OTS1 is required for maintaining gene silencing, and SUMO E3 ligase, SIZ1, is also involved in silencing regulation (Liu et al., 2017b). However, little research has been carried out yet specifically examining the role of *Arabidopsis* SUMO mutants on the nucleus.

Additionally, when a cell is stressed, many nuclear-associated proteins are SUMOylated; when the stress is removed, the proteins become deSUMOylated again, returning to normal levels, enabling rapid reversible response to differing environmental stimuli. This SUMO response is also capable of displaying memory; if a second stress is induced immediately following, a stress the stress-induced SUMOylation pattern is suppressed (Kurepa et al., 2003; Miller et al., 2013). However, it is not yet understood how the SUMO system is capable of displaying memory, and this requires additional research.

However, with advances in knowledge and technology, the ability to extract and identify SUMOylated proteins may show that SUMO has a role in many aspects of the cell and is not as limited to the nucleus. Indeed, some SUMO proteases may specifically localize to other organelles in the cell.

## Concluding Remarks

Since its discovery over 20 years ago, the role of SUMO in many different processes in plants has been uncovered. The role of SUMO in so many important aspects of plant biology, from hormonal processes, to abiotic responses to disease responses has demonstrated that SUMO is a critical post-translational modification in plants. In particular, the role of SUMO proteases has suggested that they play a vital role in regulating the SUMO cycle, making their role in plants highly critical. It is likely that more SUMO proteases will be identified with roles in numerous other pathways and in other components of the plant cell. With the identification of more SUMO proteases, it may help answer the big question in SUMO: how the SUMO system identifies targets and provides the specificity in SUMOylation. The specificity in the SUMO system may come from SUMO proteases providing specificity in deSUMOylation. With research continually uncovering important SUMOylated proteins in plants, further research is still required to understand how the SUMO proteases, through their combined action help regulate and maintain the plant SUMOylome to ensure proper growth, development, and appropriate stress responses.

## Author Contributions

RM and AS designed and wrote the article.

## Conflict of Interest Statement

The authors declare that the research was conducted in the absence of any commercial or financial relationships that could be construed as a potential conflict of interest.

## Abbreviations

PTM, post translational modification; STUbL, SUMO-targeted ubiquitin ligases; SIM, SUMO-interacting motif; SAE1/2, SUMO-activating enzyme 1/2 ; SCE1, SUMO-conjugating enzyme 1; SIZ1, SAP and MIZ1; HPY2, high ploidy 2; PIAL1, protein inhibitor of activated STAT-like 1; ULP1, ubiquitin-like protease 1; UBL, ubiquitin like; DeSI, DeSUMOylating isopeptidase; USPL1, ubiquitin-specific protease-like 1; ESD4, early in short days 4; FLC, flowering locus C; SENP1, sentrin-specific protease 1; NEM, N-ethylmaleimide; ELS1/2, ESD4-like SUMO protease 1/2; FUG1, fourth ULP gene 1; OTS1/2, overly tolerant to salt 1/2; RanGAP, Ran GTPase-activating protein; ScPCNA, proliferating cell nuclear agent; SPF1, SUMO protease related to fertility 1; ASP1, Arabidopsis SUMO protease 1; WT, wild type; PPPDE, peptidases-permuted papain fold peptidases; dsRNA, double stranded RNA; BZEL, BTB-ZF protein expressed in effector lymphocytes; FLS2, flagellin sensitive 2; UBP6, ubiquitin-specific protease 6; CAM, Calmodulin2; RGA, repressor of ga1-3; GAI, gibberellin-insensitive; GA, gibberellins; JA, jasmonic acid; SA, salicylic acid; ICS1, isochorisate synthase 1; ABA, abscisic acid; ABI5, ABA-insensitive 5; MYB30, MYB domain protein 30; JAZ6, jasmonate-ZIM-domain protein 6; COI1, coronatine-insensitive 1; GID1, gibberellin-insensitive dwarf 1; SLY1, sleepy 1; SCF, Skp, CULLIN, F-box; X.c.v., X. campestris pv. Vesicatoria; PAMP, pathogen-associated molecular patterns; BIK1, botrytis-induced kinase 1; Flg22, flagellin22; ROS, reactive oxygen species; MAPK, mitogen-activated protein kinases; Pst DC3000, Pseudomonas syringae pv. Tomato; PR1, pathogenesis-related 1; ARF7, auxin response factor 7; IAA3, indole-3-acetic acid inducible3; PEG, polyethylene glycol; OsbZIP, basic leucine zipper domain; PHYB, phytochrome B; PIF, phytochrome-interacting factor; CCA1, circadian clock-associated 1; DUBs, deubiquitinating enzymes.
